# Clinical characteristics and prognostic factors of elderly patients with coronary artery disease

**DOI:** 10.3389/fcvm.2026.1701042

**Published:** 2026-06-25

**Authors:** Shutong Wang, Chen Li, Zichen Huang, Rong Li

**Affiliations:** 1 Department of Geriatrics, Xijing Hospital, The Fourth Military Medical University, Xi’an, China; 2 Department of Geriatrics, General Hospital of Northern Theater Command, Shenyang, China

**Keywords:** coronary artery disease, elderly, OPT-CAD, prognosis, risk score model

## Abstract

**Objective:**

We investigated the clinical characteristics of elderly patients with CAD, analyzed 1-year prognostic factors, and evaluated the predictive value of the Optimal Antiplatelet Therapy for Chinese patients with Coronary Artery Disease (OPT-CAD) score in their long-term prognosis.

**Methods:**

In this cross-sectional study, 4,836 patients with coronary artery stenosis of 50% or greater aged ≥60 years were included to compare differences in clinical characteristics between the non-elderly group (*n* = 3,970, aged 60–74 years) and the elderly group (*n* = 866, aged ≥75 years). In the subsequent retrospective cohort study, a subset of 508 patients from the elderly group was followed up for 1 year (with 493 completing follow-up) to analyze risk factors affecting prognosis. The predictive performance of the OPT-CAD score was evaluated by comparing it with the Global Registry for Acute Coronary Events (GRACE) score.

**Results:**

Elderly patients exhibited lower male prevalence, lipid levels, and BMI but more extensive and severe coronary lesions. Among elderly patients, 10.3% (*n* = 51) experienced major adverse cardiovascular event (MACE), with 3.2% mortality (*n* = 16). A multivariate analysis identified anemia (HR = 2.03), left main lesion (HR = 1.93), multivessel disease (HR = 4.73), and left ventricular ejection fraction (LVEF) < 50% (HR = 1.98) as independent MACE predictors. The OPT-CAD score demonstrated moderate predictive capacity (AUC=0.628, 95% CI 0.545–0.711, *P* = 0.003) and was comparable to GRACE in the subgroup analysis (AUC=0.625 vs. 0.632, *P* = 0.878).

**Conclusion:**

In elderly patients (≥75 years old), cardiovascular risk factors such as male sex, smoking, lipid profile, and BMI were reduced and could be used to predict ischemic event risk in elderly patients with acute coronary syndrome. Anemia, LM lesion, multivessel coronary artery disease, and low ejection fraction (LVEF < 50%) were independent risk factors for adverse events in elderly patients 1 year after discharge.

## 1 Introduction

China has entered a period of accelerated aging, and the proportion of older people (aged ≥5 years) (1) is increasing. By the end of 2020, the number of people over 75 years was 67 million, accounting for 4.76% of the total population (2). Coronary artery disease (CAD) is a major threat to health (3). CAD mortality increases significantly with age and the rate rises approximately exponentially after 60 (2). Elderly patients are a ''long-lived" group of patients with CAD, in whom coronary artery lesions are complex and combined with a variety of diseases (atrial fibrillation, hypertension, diabetes, chronic kidney disease, respiratory diseases, etc.), accompanied by weakness and organ dysfunction, which lead to many challenges for clinical diagnosis and treatment.

In elderly patients, attention should be paid to the long-term management of the disease to effectively identify high-risk patients, actively delay disease progression, and improve long-term prognosis with drug therapy or revascularization. Relevant guidelines unanimously emphasize that the prognostic risk of patients should be accurately stratified according to the risk assessment system. The Optimal Antiplatelet Therapy for Chinese patients with Coronary Artery Disease (OPT-CAD) score is mainly used to predict the long-term ischemic risk of patients with CAD after discharge and provide a decision-making basis for the individualized treatment of local patients in China. It consists of 10 independent risk factors: age, heart rate, hypertension, prior myocardial infarction, prior stroke, renal insufficiency, anemia, low ejection fraction (LVEF < 50%), positive cardiac troponin level, and ST-segment deviation. The developers of the OPT-CAD score assert that it has clinical utility in predicting 1-year ischemic events and all-cause mortality. However, its use in the elderly has rarely been reported. This study aimed to summarize the clinical characteristics of elderly patients with CAD, analyze the predictive value of the OPT-CAD score on the risk of ischemia in elderly patients through follow-up, compare it with a well-validated score [Global Registry for Acute Coronary Events (GRACE)] (4), investigate the main factors affecting prognosis, and provide a reference for disease management and treatment decisions in the growing population of elderly patients with CAD.

## 2 Materials and methods

### 2.1 Patient population and study design

**Figure 1:**
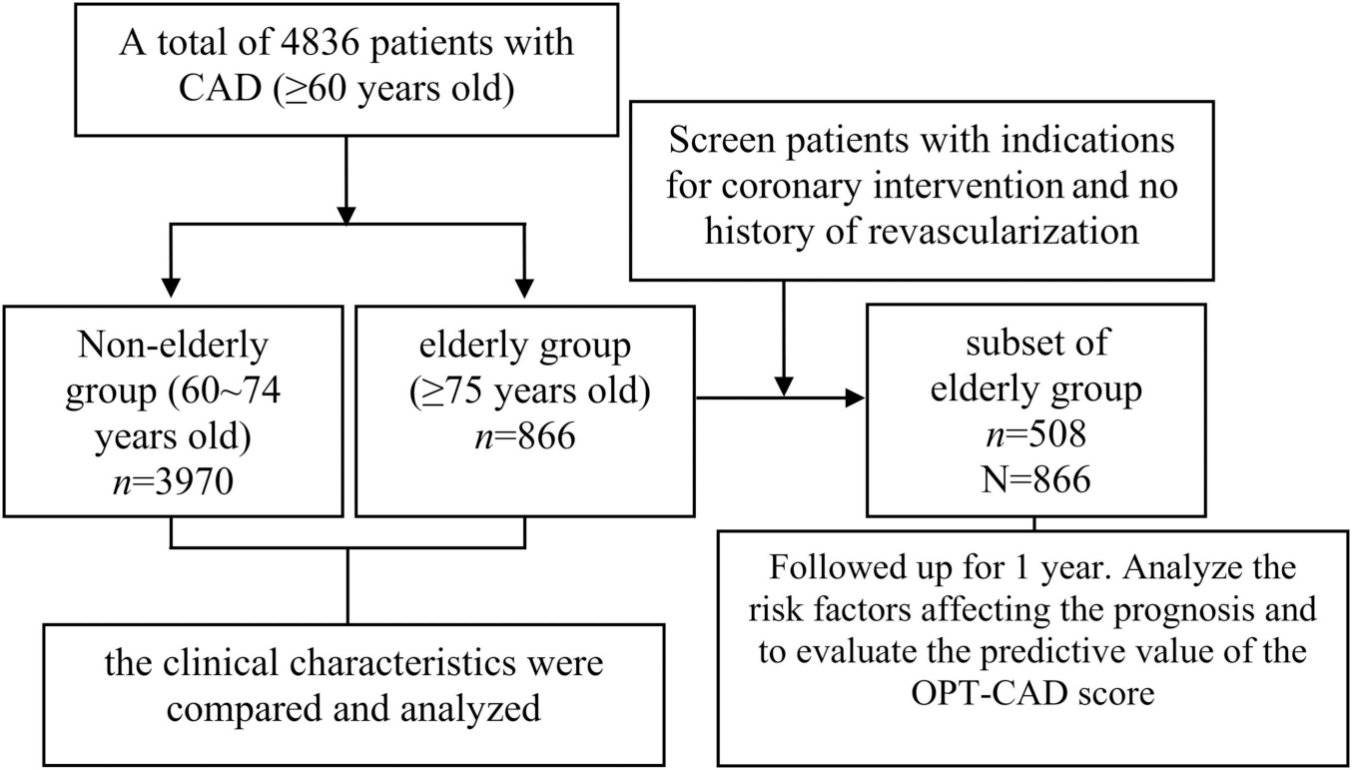
Flowchart of the study.

First, among 4,836 individuals with coronary stenosis ≥50%, differences in clinical risk factors were compared between the age group of 60–74 years (*n* = 3,970) and those aged ≥75 years (*n* = 866) in the cross-sectional study. The inclusion criteria were: (1) invasive coronary angiography performed at Xijing Hospital, Fourth Military Medical University between January 2019 and December 2020, showing stenosis ≥50% in at least one coronary artery; (2) age ≥60 years. The exclusion criteria were: (1) history of self-reported severe mental disorders (e.g., major depression), malignancy, or serious infectious diseases (e.g., severe pneumonia); and (2) missing data with no possibility of patient contact. Subsequently, a retrospective cohort study was conducted to explore prognostic factors for 1-year major adverse cardiovascular events among individuals aged ≥75 years with coronary stenosis ≥70% and persistent myocardial ischemic symptoms despite medical therapy (*n* = 508). This study was approved by the Ethics Committee of Xijing Hospital, Fourth Military Medical University (approval no. KY20222043-C-1). Given the observational nature of the study—no intervention was imposed on participants and no risk of personal privacy disclosure existed—the committee waived the requirement for informed consent (Figure 1).

### 2.2 Methods

Baseline characteristics, laboratory test results, transthoracic echocardiography, and invasive coronary angiography results of the enrolled patients were collected using an electronic medical record system. Based on coronary angiography and surgical records, the location of coronary artery lesions and the degree of stenosis in patients were collected, and the Gensini score was used to quantitatively evaluate the degree of coronary artery lesions (5). The OPT-CAD score was used to calculate the prognostic score of the follow-up patients (6), and a GRACE score of 2.0 was applied to patients with acute coronary syndrome (ACS). Endpoint events were defined as major adverse cardiovascular events (MACE) during the follow-up period. MACE is defined as a composite endpoint that includes cardiovascular death, non-fatal myocardial infarction, non-fatal stroke, or any revascularization (7). During outpatient or telephonic follow-up, patients were asked whether they had experienced any MACE within 1 year after undergoing invasive coronary angiography. All events were recorded by a cardiologist after individual determinations.

### 2.3 Statistical methods

SPSS Statistics software (version 26.0) was used for statistical analyses. The measurement data conforming to the normal distribution were described by mean ± standard deviation ($\bar{x}\pm s$) and Student's t-test. Data that did not have a normal distribution were represented by the median (quartile) [M (P_25_, P_75_)] and analyzed using the Mann–Whitney *U* test. The enumeration data were expressed as frequency and rate (%), and the *χ*^2^ test or Fisher's exact probability method was used for statistical analysis. The Kaplan–Meier method and log-rank test were used for survival analysis. The proportional hazards assumption was evaluated using the test of Schoenfeld residuals. Under the premise that all variables met the proportional hazards assumption, the COX regression model was used to evaluate the association of clinical factors with endpoint events. The area under the receiver operating characteristic curve (AUROC) was used to assess the predictive value and compared using the *Z*-test. All tests were two-sided, and differences were considered statistically significant at *P* < 0.05.

## 3 Results

### 3.1 Comparison of major cardiovascular risk factors between patients from the elderly and non-elderly groups

In total, 4,836 patients with CAD (3,970 in the non-elderly group and 866 in the elderly group) were included. Ccardiovascular risk factors such as male sex, lipid profile, and body mass index (BMI) were reduced in the elderly group (*P* < 0.01), and the proportion of patients with hypertension and systolic blood pressure on admission was higher than that in the non-elderly group (*P* < 0.01). There was no significant difference in the prevalence of type 2 diabetes between the two groups (*P* > 0.05). In terms of clinical features, previous percutaneous coronary intervention (PCI), stroke history, ST-segment elevation myocardial infarction (STEMI), non-ST-segment elevation myocardial infarction (NSTEMI), and left ventricular ejection fraction (LVEF) < 50% were more common in the elderly group than in the non-elderly group (*P* < 0.05). The proportion of patients with atrial fibrillation was lower in the elderly than in the non-elderly group (*P* < 0.01). In terms of laboratory examinations, hemoglobin and triglyceride (TG) levels were lower in the elderly group than in the non-elderly group (*P* < 0.01), and high-density lipoprotein cholesterol (HDL-C) levels were higher in the elderly group than in the non-elderly group (*P* < 0.01). There were no significant differences in total cholesterol (TC), low-density lipoprotein cholesterol (LDL-C), or fasting blood glucose (FBG) levels between the two groups (*P* > 0.05) (Table 1).

**Table 1:** Baseline characteristics of patients of the elderly and non-elderly groups.

Variable	Non-elderly group (*n* = 3,970)	Elderly group (*n* = 866)	*P*-value
Age, years	66.4 ± 4.0	78.9 ± 3.6	<0.001
Men	2,810 (70.8)	556 (64.2)	<0.001
BMI (kg/m^2^)	24.7 ± 3.0	23.8 ± 3.2	<0.001
Hypertension	2,290 (57.7)	541 (62.5)	0.010
Diabetes mellitus	1,192 (30.0)	235 (27.1)	0.091
History of smoking	1,628 (41.0)	267 (30.8)	<0.001
Previous PCI	1,063 (26.8)	268 (30.9)	0.007
Previous MI	478 (12.0)	112 (12.9)	0.467
Previous stroke	478 (12.0)	124 (14.3)	<0.001
Atrial fibrillation	351 (8.8)	39 (4.5)	0.008
STEMI	219 (5.5)	65 (7.5)	0.024
NSTEMI	96 (2.4)	33 (3.8)	0.021
SBP (mmHg)	133.1 ± 20.5	136.9 ± 22.0	<0.001
DBP (mmHg)	72.9 ± 11.7	70.2 ± 12.7	<0.001
LVEF<50%	430 (10.8)	126 (14.5)	0.002
Hemoglobin (g/L)	141.3 (130.4, 151.2)	135.0 (122.5, 147.1)	<0.001
TC (mmol/L)	3.5 (3.0, 4.2)	3.6 (3.0, 4.2)	0.866
TG (mmol/L)	1.3 (0.9, 1.7)	1.1 (0.8, 1.6)	<0.001
LDL-C (mmol/L)	1.9 (1.5, 2.6)	1.9 (1.5, 2.5)	0.202
HDL-C (mmol/L)	1.0 (0.9, 1.2)	1.1 (0.9, 1.3)	<0.001
FBG (mmol/L)	5.6 (5.0, 7.3)	5.7 (5.0, 7.2)	0.753

Values are expressed as mean ± SD, *n* (%) or [M (P25, P75)]. LVEF was measured using transthoracic echocardiography by the biplane Simpson method. BMI, body mass index; PCI, percutaneous coronary intervention; MI, myocardial infarction; STEMI, ST-segment-elevation myocardial infarction; NSTEMI, non-ST-segment-elevation myocardial infarction; SBP, systolic blood pressure; DBP, diastolic blood pressure; LVEF, left ventricular ejection fraction; TC, total cholesterol; TG, triglyceride; LDL-C, low-density lipoprotein cholesterol; HDL-C, high-density lipoprotein cholesterol; FBG, fasting blood glucose.

### 3.2 Coronary artery lesions

Compared with the non-elderly group, the elderly group exhibited a higher prevalence of left main (LM) disease, right coronary artery (RCA) disease, and multivessel disease (MVD) (*P* < 0.05). No significant differences were observed between the two groups regarding the presence of lesions in the left anterior descending artery (LAD) or the left circumflex artery (LCX) (*P* > 0.05). The severity of coronary artery disease was quantitatively assessed using the Gensini score, which was significantly higher in the elderly group than in the non-elderly group (*P* = 0.002) (Table 2).

**Table 2:** Lesion branches and degree of coronary artery stenosis.

Variable	Non-elderly group(*n* = 3,970)	Elderly group(*n* = 866)	*P*-value
Presence of lesion, *n* (%)			
LM	418 (10.5)	124 (14.3)	0.001
LAD	3,245 (81.7)	717 (82.8)	0.464
LCX	2,439 (61.4)	550 (63.5)	0.255
RCA	2,419 (60.9)	572 (66.1)	0.005
MVD	1,467（37.0）	368（42.5）	0.002
Gensini score	41.8 (21.8, 72.0)	48.3 (22.8, 84.0)	0.002

Values are expressed as *n* (%) or [M (P25, P75)]. LM, left main coronary artery; LAD, left anterior descendant; LCX, left circumflex branch; RCA, right coronary artery; MVD, multivessel coronary artery disease.

### 3.3 High-risk factors for MACE

Furthermore, 508 elderly patients with indications for coronary intervention and no history of revascularization were screened for a 1-year follow-up, and 493 (97.0%) patients completed the study. Of the primary outcomes, 51 patients (10.3%) had MACE and 16 patients (3.2%) had all-cause deaths. STEMI, LVEF < 50%, and LM lesions were more common in deceased patients compared with living patients (*P* < 0.05). In terms of MACE, compared with patients in whom MACE did not occur, the hemoglobin level was lower (*P* < 0.01), and the LM lesions, MVD, and LVEF < 50% were higher (*P* < 0.01) (Table 3).

**Table 3:** Baseline characteristics of patients from different clinical outcomes.

Variable	MACE		
	Occurred (*n* = 51)	Not occurred (*n* = 442)	*P*-value
Age, years	79.00 ± 3.35	78.58 ± 3.24	0.382
OPT-CAD score	118.88 ± 35.27	104.24 ± 31.25	0.002
Men, *n* (%)	37 (72.5)	272 (61.5)	0.124
BMI, kg/m^2^	23.87 ± 3.31	23.76 ± 3.14	0.817
Hypertension (%)	31 (60.8)	281 (63.6)	0.695
Diabetes mellitus, *n* (%)	14 (27.5)	106 (24.0)	0.585
History of smoking, *n* (%)	14 (27.5)	142 (32.1)	0.497
Previous PCI *n* (%)	3 (5.9)	27 (6.1)	0.949
Previous stroke, *n* (%)	5 (9.8)	76 (17.2)	0.177
Atrial fibrillation, *n* (%)	4 (7.8)	18 (4.1)	0.381
NSTEMI, *n* (%)	6 (11.8)	24 (5.4)	0.138
STEMI, *n* (%)	7 (13.7)	53 (12.0)	0.720
UAP, *n* (%)	30 (58.8)	299 (67.6)	0.205
ACS, *n* (%)	43 (84.3)	376 (85.1)	0.886
LVEF < 50%	15 (29.4)	59 (13.3)	0.002
Hemoglobin (g/L)	127.22 ± 20.79	136.16 ± 19.04	0.002
TC (mmol/L)	3.75 ± 0.92	3.78 ± 0.98	0.808
TG (mmol/L)	1.19 ± 0.56	1.43 ± 1.60	0.278
LDL-C (mmol/L)	2.21 ± 0.75	2.13 ± 0.81	0.495
HDL-C (mmol/L)	1.05 ± 0.22	1.14 ± 0.30	0.045
FBG (mmol/L)	6.65 ± 2.79	6.67 ± 2.97	0.955
Presence of lesion, *n* (%)			
LM	15 (29.4)	64 (14.5)	0.006
LAD	47 (92.2)	400 (90.5)	0.895
LCX	41 (80.4)	280 (63.3)	0.016
RCA	43 (84.3)	316 (71.5)	0.051
MVD	49 (96.1)	345 (78.1)	0.002
Current PCI, *n* (%)	42 (82.4)	365 (82.6)	0.968

LVEF was measured using transthoracic echocardiography by the biplane Simpson method. CCS, chronic coronary syndromes.

Under the premise that all variables met the proportional hazards assumption (all *P* > 0.05), a multivariate COX regression analysis was performed using MACE as the dependent variable. The results showed that LVEF < 50% (HR = 1.98; 95% CI 1.07–3.67; *P* = 0.029), anemia (HR = 2.03; 95% CI 1.06–3.87; *P* = 0.032), LM lesions (HR = 1.93; 95% CI 1.05–3.55; *P* = 0.036), and MVD (HR = 4.73; 95% CI 1.13–19.82; *P* = 0.034) were independent risk factors for 1-year MACE in elderly patients with CAD (Table 4).

**Table 4:** COX regression analysis of prognostic factors for MACE.

Variable	Univariate analysis		Multivariate analysis	
	HR (95% CI)	*P-*value	HR (95% CI)	*P*-value
Age, years	1.08 (0.69–1.70)	0.737		
Female sex	0.62 (0.30–1.26)	0.186		
Hypertension	1.10 (0.59–2.04)	0.782		
Diabetes mellitus	1.05 (0.72–1.52)	0.819		
History of smoking	0.73 (0.33–1.62)	0.439		
Previous MI	0.65 (0.18–2.31)	0.505		
Previous stroke	0.47 (0.17–1.26)	0.132		
Atrial fibrillation	1.94 (0.60–6.30)	0.268		
CCS	1.48 (0.43–6.12)	0.537		
ACS				
UAP	1.50 (0.46–4.84)	0.505		
NSTEMI	3.17 (0.79–12.75)	0.104		
STEMI	1.07 (0.34–3.40)	0.903		
LVEF < 50%	2.52 (1.24–5.13)	0.011	1.98 (1.07–3.67)	0.029
Anemia	2.25 (1.11–4.61)	0.025	2.03 (1.06–3.87)	0.032
Elevated TC	0.18 (0.02–1.65)	0.129		
Elevated TG	1.09 (0.29–4.09)	0.896		
Elevated LDL-C	2.46 (0.66–9.24)	0.181		
Decreased HDL-C	1.05 (0.20–5.55)	0.957		
Elevated FBG	0.97 (0.48–1.94)	0.926		
Presence of lesion				
LM	1.81 (0.89–3.67)	0.102	1.93 (1.05–3.55)	0.036
LAD	0.82 (0.20–3.32)	0.776		
LCX	1.44 (0.43–4.86)	0.557		
RCA	1.11 (0.24–5.18)	0.896		
MVD	4.46（0.63–31.62）	0.135	4.73（1.13–19.82）	0.034
Current PCI	0.70 (0.32–1.53)	0.372		

LVEF was measured using transthoracic echocardiography by the biplane Simpson method. Anemia was defined as a hemoglobin level of <120 g/L in males and <110 g/L in females.

A subgroup analysis showed that LVEF < 50% (HR = 2.13; 95% CI: 1.07–4.23; *P* = 0.032), LM lesion (HR = 2.48; 95% CI 1.29–4.77; *P* = 0.007), and anemia (HR = 3.02; 95% CI 1.54–5.92; *P* = 0.001) were independent risk factors for MACE in patients with ACS. LVEF < 50% (HR = 2.26; 95% CI 1.17–4.36; *P* = 0.015), LM lesion (HR = 2.37; 95% CI 1.23–4.57; *P* = 0.010), and anemia (HR = 2.50; 95% CI 1.26–4.98; *P* = 0.009) were independent risk factors for 1-year MACE in patients undergoing PCI. No corresponding independent risk/protective factors were found in the chronic coronary syndromes (CCS) subgroup and non-PCI subgroups after the multivariate COX regression analysis (Table 5).

**Table 5:** Subgroup analysis of multivariate COX regression for MACE.

Subgroup	*n* (%)	LVEF < 50%	LM lesion	Anemia
CCS	74 (15.0%)	3.619 (0.870–15.045)	1.846 (0.371–9.180)	0.415 (0.480–3.566)
		*P* = 0.077	*P* = 0.454	*P* = 0.423
ACS	419 (85.0%)	2.126 (1.068–4.234)	2.478 (1.288–4.769)	3.022 (1.542–5.924)
		*P* = 0.032	*P* = 0.007	*P* = 0.001
Non-PCI	86 (17.4%)	1.270 (0.159–10.176)	2.272 (0.471–10.955)	0.977 (0.122–7.812)
		*P* = 0.822	*P* = 0.307	*P* = 0.982
PCI	407 (82.6%)	2.260 (1.172–4.360)	2.369 (1.227–4.574)	2.503 (1.259–4.976)
		*P* = 0.015	*P* = 0.010	*P* = 0.009

LVEF was measured using transthoracic echocardiography by the biplane Simpson method.

### 3.4 Evaluation of the OPT-CAD score in elderly patients

**Figure 2:**
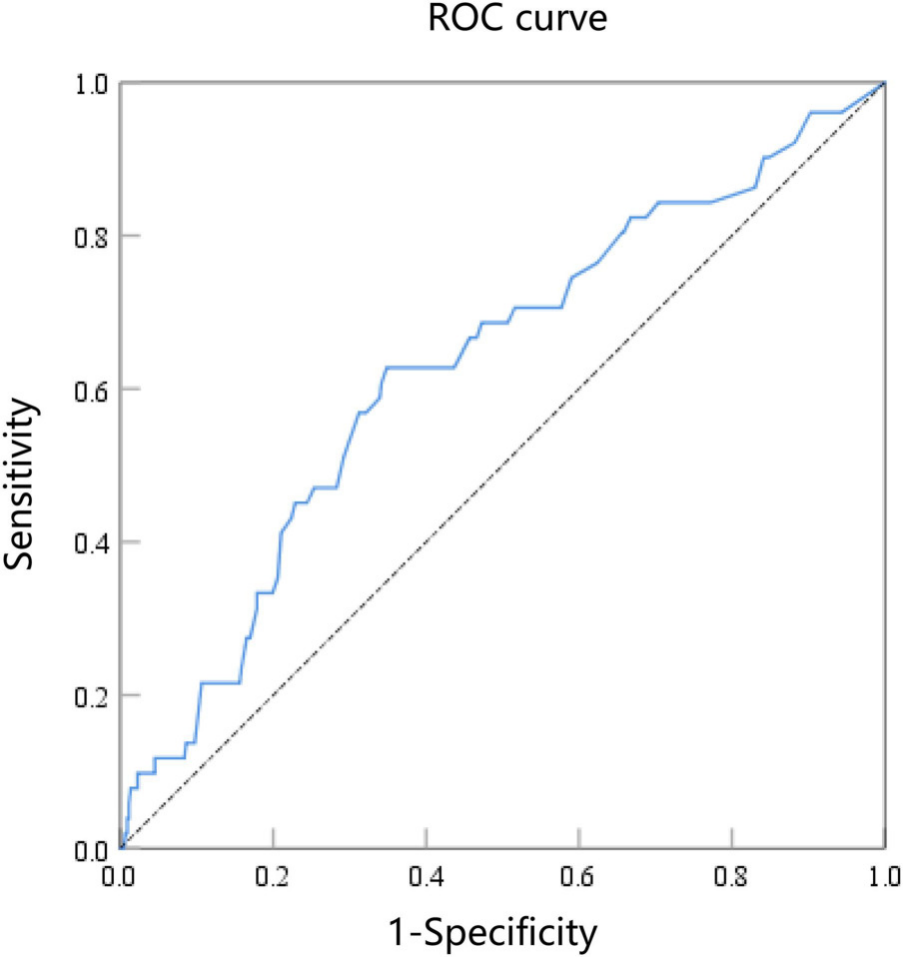
Receiver operating characteristic (ROC) curves for the OPT-CAD score to predict MACE in 1 year for all patients in the follow-up group. (AUROC=0.628; 95% CI 0.545-0.711).

**Figure 3:**
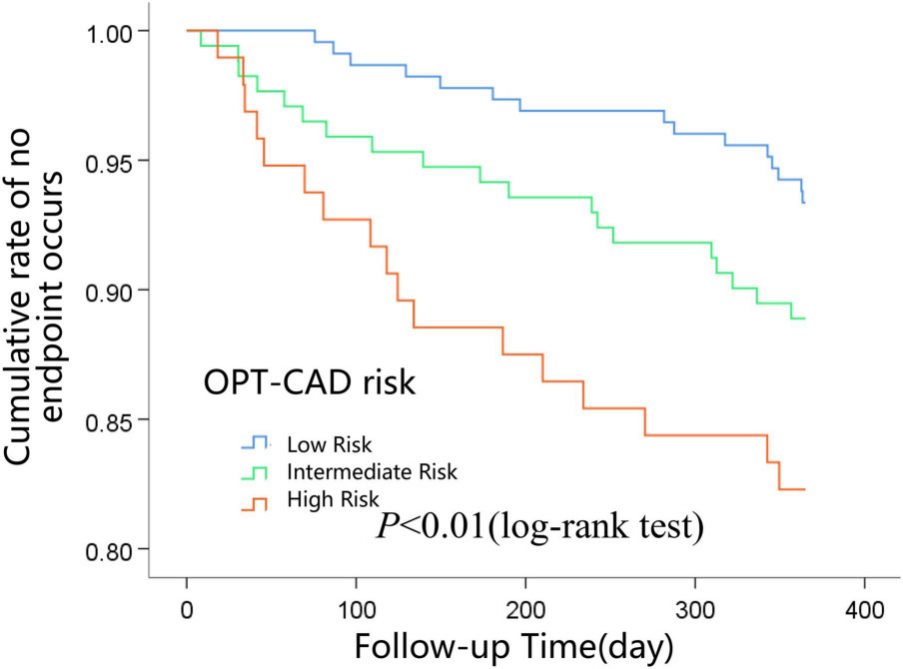
Observed rates of ischemic events at 1-year follow-up among elderly patients by OPT-CAD score.

The predictive value of the OPT-CAD score for the outcome of MACE in the entire follow-up group (AUROC=0.628; 95% CI 0.545–0.711; *P* = 0.003) was moderate (Table 6, Figure 2). The survival curve was plotted according to the risk stratification of the OPT-CAD score. There was a significant difference between the high, intermediate, and low groups at the endpoint of MACE (*P* < 0.01) (Figure 3).

**Table 6:** AUROC of the OPT-CAD score for MACE.

Group	*n* (%)	AUROC (95% CI)	*P*-value
Total cases	493	0.628 (0.545–0.711)	0.003
*Subgroup*			
CCS	74 (15.0%)	0.638 (0.456–0.821)	0.204
ACS	419 (85.0%)	0.625 (0.533–0.717)	0.007
UAP	329 (66.7%)	0.602 (0.487–0.717)	0.066
NSTEMI	64 (13.0%)	0.576 (0.384–0.769)	0.436
STEMI	60 (12.2%)	0.716 (0.539–0.892)	0.017
Non-PCI	86 (17.4%)	0.543 (0.329–0.756)	0.677
PCI	407 (82.6%)	0.647 (0.559–0.734)	0.002

**Figure 4:**
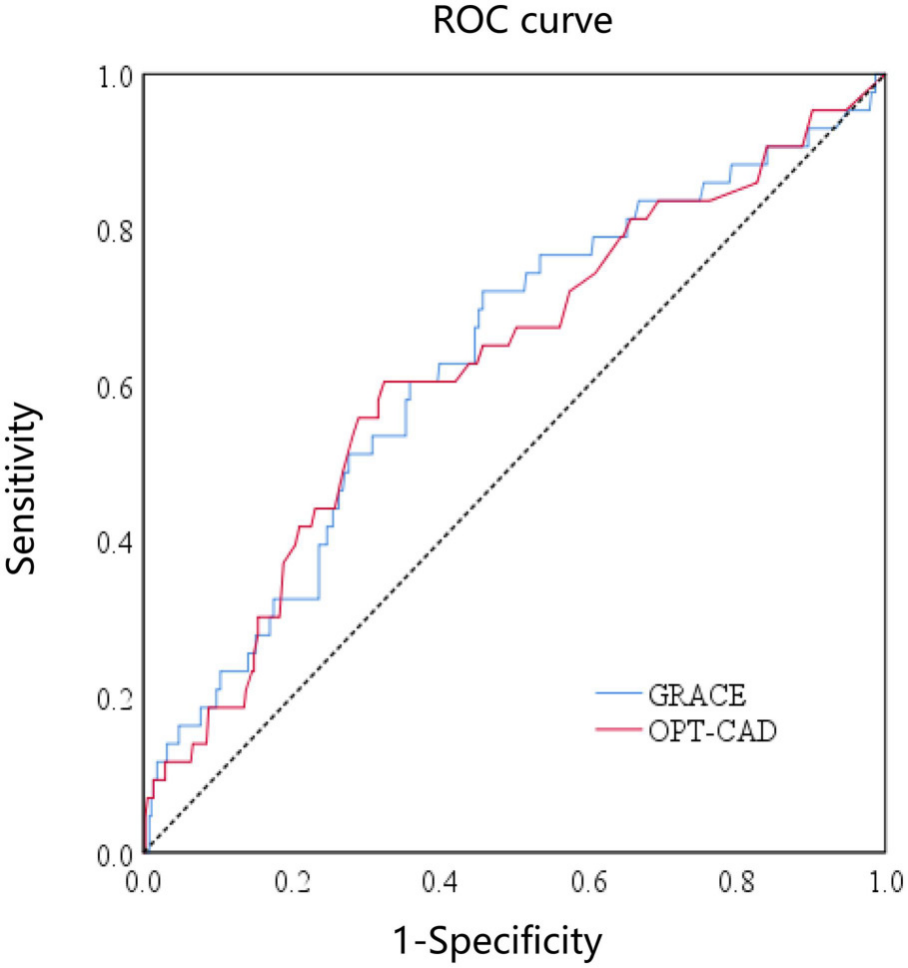
ROC curves for OPT-CAD and GRACE scores to predict MACE in 1 year for the group of elderly patients with ACS. OPT-CAD (AUROC=0.625; 95% CI 0.533–0.717); GRACE (AUROS=0.632; 95% CI 0.541–0.724).

The subgroup analysis showed predictive value for identifying the endpoint of MACE in patients who underwent PCI (AUROC=0.647; 95% CI 0.559–0.734; *P* = 0.002). In the ACS group, the OPT-CAD score also showed predictive value (AUROC=0.625; 95% CI 0.533–0.717; *P* = 0.007). Compared with the GRACE score (AUROC=0.632; 95% CI 0.541–0.724; *P* = 0.005), there was no statistically significant difference (*P* = 0.878) (Table 6, Figure 4). However, the predictive value was not satisfactory for the outcomes of patients with CCS (AUROC=0.638; 95% CI 0.456–0.821; *P* = 0.204) or those who underwent pharmacotherapy (AUROC=0.543; 95% CI 0.329–0.756; *P* = 0.677) (Table 6).

## 4 Discussion

With an aging population and changes in diet and lifestyle, the risk factors of patients with CAD in China have undergone tremendous changes. At the same time, with the development of coronary revascularization technology and the application of more effective cardiovascular drugs, the effectiveness of existing prediction models may improve. However, previous studies have had the problem of a relatively small number of elderly patients and a relative lack of data on patients of advanced age (≥75 years). To address the increasing prevalence of CAD in advanced-age populations, studies specifically targeting advanced-age patients are required to provide reliable evidence supporting the selection of appropriate treatment strategies.

Our study showed that elderly patients had a higher coronary Gensini score; a higher proportion of LM, RCA, and MVD lesions; more extensive coronary lesions; and more severe stenosis. It is well known that with increasing age, the influence of multiple risk factors on the structure and metabolism of the arterial wall continues to grow, the function of endothelial cells gradually declines, and the arterial wall will generally show thickening of the intima and decreasing vascular elasticity. Nevertheless, we found that risk factors such as male sex, smoking status, BMI, lipid profile, and other CAD risk factors were lower in elderly patients. The incidence of cardiovascular disease is positively correlated with age, and studies have shown that this correlation is partly due to the cumulative effects of risk factors throughout life. Risk factors such as blood pressure, cholesterol, and diabetes increase with age (8). However, data analysis based on multicountry health surveys has shown that although the risk factors for CAD and age change over time, the relationship between these risk factors and age increases in people over 65 years first flattens and then reverses to an inverse correlation, that is, the level of risk factors in older people decreases (9). This finding is consistent with the results of the present study.

The reasons for this outcome may be that people with high-risk factor levels tend to have faster progression, poorer prognosis, and greater probability of early death and therefore often cannot enter the elderly stage. Therefore, as elderly patients with CAD are a relatively “long-lived” group, we should recognize the complexity of these patients, conduct adequate examinations and evaluations, and carry out individualized treatment interventions.

In older adults, the association between traditional risk factors such as low-density lipoprotein cholesterol and cardiovascular mortality weakens, sometimes even exhibiting a “protective” illusion. The underlying reason lies in the fact that individuals with higher levels of these risk factors tend to experience cardiovascular events at younger ages and are less likely to survive into old age; consequently, those who enter an elderly cohort are essentially “survivors,” with their risk factor profiles having undergone a selective shift. Furthermore, with advancing age, the pathophysiological mechanisms of lipid metabolism, inflammatory pathways, and vascular remodeling may change, and the effect size of risk factors on endpoints becomes modified by other age-related risk factors (e.g., frailty and comorbidity)—a phenomenon known as effect modification (10). A community-based study of individuals aged over 70 years also confirmed that systolic blood pressure, total cholesterol, and HDL cholesterol no longer possess traditional predictive value in the elderly (11). Therefore, the features observed in the older patients with CAD in this study—namely low BMI, low lipid profile, and a lower proportion of men—should be interpreted as characteristics of “selective survival” in this specific population, rather than a true “reversal” of risk factors.

We found that LVEF < 50%, anemia, and LM/MVD lesions were independent risk factors for MACE in elderly patients with CAD 1 year after discharge. Left ventricular insufficiency is a strong predictor of the long-term prognosis in patients (12). LVEF is a critical indicator for evaluating cardiac function, and the degree of heart failure can be assessed using the ejection fraction (13). For elderly patients, the evaluation of cardiac function is more important. Based on a Chinese patient cohort, the OPT-CAD study found that an LVEF < 50% was valuable in predicting long-term ischemic events after discharge (6), which is consistent with the results of this study.

The LM is a crucial vascular channel in the coronary artery. Once a lesion occurs, it has a wide range of effects, often accompanied by diffuse or long lesions, and whether conservative treatment or PCI is performed, its prognosis is poor (14). MVD is more common in elderly patients and is a critical risk factor for long-term adverse events (15).

Hemoglobin is an essential component of blood cells and plays a vital role in oxygen transport in the body. Increased hemoglobin concentrations have been shown to significantly reduce the risk of death, and high hemoglobin levels are protective factors in patients with heart failure (16). In addition, various studies have shown that low hemoglobin levels are strongly associated with poor prognosis in patients with CAD (17, 18). Elderly patients have attenuated physical function, and once anemia occurs, the myocardial tissue is often in a state of insufficient oxygen supply for a long time, which is more likely to induce cardiovascular adverse events.

The identification of the above independent risk factors carries important implications for the management of elderly patients with coronary artery disease (CAD). With regard to anemia, our findings showed that anemia was associated with an approximately twofold increase in 1-year major adverse cardiovascular events (MACE), which is highly consistent with a recent study by Jonik et al. (19) published in 2024. In that study of 679 patients with multivessel disease undergoing PCI over a 6-year follow-up, the all-cause mortality rate was 20.0% in the anemic group, significantly higher than 11.6% in the non-anemic group (*P* = 0.003); anemia was also associated with higher rates of myocardial infarction and in-hospital mortality. Li et al. (20) further demonstrated that anemia was an independent risk factor for worsening renal function and death in elderly patients with CAD. The clinical implication is that for elderly patients with CAD with concomitant anemia, active investigation of the underlying causes (e.g., malnutrition, chronic kidney disease, and occult gastrointestinal bleeding) is warranted, along with optimized nutritional support. Moreover, a careful balance between bleeding and ischemic risks should be considered when administering antithrombotic therapy to avoid exacerbating anemia due to intensified antithrombotic regimens. The prognostic significance of left main (LM) disease combined with MVD is even more pronounced. In the present study, MVD was associated with a nearly four-fold increase in MACE risk and LM disease with an approximately twofold increase. The left main coronary artery supplies 75% of left ventricular blood flow; once symptomatic, LM disease carries a mortality rate exceeding 30% within two years. When LM disease coexists with MVD and an LVEF <50%, cardiac function is already on the verge of decompensation. Such cases were historically considered a “no-go zone” for cardiac surgery. However, with recent advances in preoperative mechanical circulatory support (e.g., intra-aortic balloon counterpulsation) and the maturation of off-pump coronary artery bypass grafting techniques, high-risk patients may still derive survival benefits from complete revascularization. Subgroup analyses in our study showed higher hazard ratios for the above risk factors in both the ACS subgroup and the PCI subgroup, further suggesting that such patients may benefit more from aggressive revascularization. Therefore, in elderly patients with low ejection fraction and complex coronary lesions, the opportunity for revascularization should not be readily dismissed; rather, an individualized strategy should be formulated by a heart team after comprehensive evaluation.

The following limitations in this study should be acknowledged. First, although our findings indicate that the above variables are independent risk factors for one-year all-cause death and major adverse cardiovascular events in older adults with coronary artery disease, multicenter randomized controlled trials are still needed to determine whether intervening on these factors could improve long-term outcomes in this population. Second, given that all participants were enrolled from a single center (Xijing Hospital), selection bias cannot be ruled out. Whether the conclusions apply to community-dwelling populations or individuals from different countries, regions, or ethnic groups requires further investigation. Third, the limited sample size and short follow-up period resulted in a small number of observed outcome events. Expanding the sample and extending follow-up would enhance statistical power. Fourth, because of the inherent constraints of a retrospective study, missing data on frailty precluded its inclusion as a confounding factor.

The OPT-CAD score was recently proposed in a prospective, real-world observational study to develop a prediction model of ischemic events in Chinese patients with CAD (6). The 10 predictors included were more objective and readily available than the other scores. Studies have shown that the OPT-CAD score performs better than the GRACE score in predicting long ischemic events in patients (6). However, this has not been well-validated or optimized for use in elderly patients, and the risk factor score associated with age increases per 10-years, which may affect its predictive accuracy in elderly patients. Our study aimed to determine the applicability of the OPT-CAD score in elderly patients with CAD aged >75 years. We found that it performed moderately in predicting 1-year MACE in elderly patients with CAD. The GRACE score is considered to be the most robust score for patients with ACS, and its online version 2.0 has been well validated, and compared with it, the OPT-CAD score showed a similar predictive value for ischemic events in the ACS subgroup (*P* = 0.878). It should be noted that, unlike the results of the OPT-CAD study (6), the OPT-CAD score did not perform better than the GRACE score in our study.

Elderly patients have higher rates of ischemic events than younger patients. Therefore, the identification of ischemic events in elderly patients is critical. In our study, among the elderly patients, the OPT-CAD score performed moderately in predicting long-term ischemic events. However, perfect performance of the OPT-CAD score was not observed in the CCS and drug therapy subgroups. This may be attributable to the fact that elderly patients with CCS often have complex lesions and may have collateral circulation compensation. In addition, the progression of coronary artery lesions may be effectively controlled after continuous and standardized treatment in the drug therapy subgroup of elderly patients. Therefore, it is difficult to accurately assess myocardial hypoxia using only conventional indicators. Although we attempted to expand the sample size, this study was ultimately based on a limited sample size of 493 patients. The performance of the OPT-CAD score in elderly patients requires real-world sample validation. In addition, as a single-center study, although the quality control of our hospital is reliable, variations may exist in certain factors, such as the assessment of coronary stenosis across different medical institutions.

This study represents the first systematic analysis of clinical characteristics and prognostic factors in Chinese patients with CAD aged ≥75 years, while also validating the potential utility of the OPT-CAD score in this population. Although its predictive performance requires further optimization, the findings provide critical insights for individualized management of elderly patients, particularly in addressing China-specific clinical challenges such as high anemia prevalence and complex coronary lesions.

## 5 Conclusion

Although coronary lesions are more extensive and complex, the levels of major cardiovascular risk factors in elderly patients with CAD without previous PCI are lower than those in younger patients. Anemia, LM lesions, MVD, and low ejection fraction (LVEF < 50%) were identified as independent risk factors for adverse events in elderly patients at 1 year postdischarge. The OPT-CAD scoring system could provide an alternative tool for predicting the risk of MACE within 1 year in elderly patients with ACS, facilitating appropriate personalized decision-making for these patients. Our findings require further confirmation in longitudinal studies and large-scale randomized trials.

## Data Availability

The raw data supporting the conclusions of this article will be made available by the authors without undue reservation.

## References

[1] Arnett DK Blumenthal RS Albert MA Buroker AB Goldberger ZD Hahn EJ. 2019 ACC/AHA guideline on the primary prevention of cardiovascular disease: a report of the American College of Cardiology/American Heart Association task force on clinical practice guidelines. Circulation. (2019) 140(11):e596–646. 10.1161/CIR.0000000000000678 30879355 PMC7734661

[2] National Bureau of statistics of China. China Statistical Yearbook-2021. Beijing: China Statistics Press (2021). p. 54.

[3] National Health Commission. 2021 China Health Statistics Yearbook. Beijing: Peking Union Medical College Press (2021). p. 282–96.

[4] Fox KA Fitzgerald G Puymirat E Huang W Carruthers K Simon T. Should patients with acute coronary disease be stratified for management according to their risk? Derivation, external validation and outcomes using the updated GRACE risk score. BMJ open. (2014) 4(2):e004425. 10.1136/bmjopen-2013-004425 24561498 PMC3931985

[5] Rampidis GP Benetos G Benz DC Giannopoulos AA Buechel RR. A guide for gensini score calculation. Atherosclerosis. (2019) 287:181–3. 10.1016/j.atherosclerosis.2019.05.012 31104809

[6] Han Y Chen J Qiu M Feng Y Meng L Sun Y. Predicting long-term ischemic events using routine clinical parameters in patients with coronary artery disease: the OPT-CAD risk score. Cardiovasc Ther. (2018) 36(5):e12441. 10.1111/1755-5922.12441 29869835

[7] Xiaoying L Miaohan Q Kun N Li X Qiu M Li Y. Comparison of the efficacy and safety of ticagrelor and clopidogrel in patients with acute coronary syndrome after risk stratification. Catheter Cardiovasc Interv. (2021) 97(Suppl 2):1032–9. 10.1002/ccd.29591 33650763

[8] Messerli HF Grodzicki T Messerli A Bangalore S Rexhaj E. Age, cardiovascular risk, and blood pressure target. J Am Coll Cardiol. (2018) 72(7):818–9. 10.1016/j.jacc.2018.05.054 30092961

[9] Singh GM Danaei G Pelizzari PM Lin JK Cowan MJ Stevens G A. The age associations of blood pressure, cholesterol, and glucose. Circulation. (2012) 125(18):2204–11. 10.1161/CIRCULATIONAHA.111.058834 22492580 PMC4174463

[10] Rozing MP Westendorp RGJ. Altered cardiovascular risk pattern of LDL cholesterol in older adults. Curr Opin Lipidol. (2023) 34(1):22–6. 10.1097/MOL.0000000000000859 36413436

[11] van Bussel EF Richard E Busschers WB Steyerberg EW van Gool WA Moll van Charante EP. A cardiovascular risk prediction model for older people: development and validation in a primary care population. J Clin Hypertens (Greenwich). (2019) 21(8):1145–52. 10.1111/jch.13617 31294917 PMC6772108

[12] Marcos-Garces V Gavara J Lopez-Lereu PM Monmeneu JV Rios-Navarro C de Dios E. Ejection fraction by echocardiography for a selective use of magnetic resonance after infarction. Circ Cardiovasc Imaging. (2020) 13(12):e011491. 10.1161/CIRCIMAGING.120.011491 33297764

[13] Rosano GMC, Teerlink JR, Kinugawa K, Bayes-Genis A, Chioncel O, Fang J, et al. The use of left ventricular ejection fraction in the diagnosis and management of heart failure. A clinical consensus statement of the Heart Failure Association (HFA) of the ESC, the Heart Failure Society of America (HFSA), and the Japanese Heart Failure Society (JHFS). Eur J Heart Fail. (2025) 27(7):1174–87. 10.1002/ejhf.3646 40260636

[14] Zafiraki VK Kosmacheva ED Shulzhenko LV Kizhvatova NV Nemtsova EA Pershukov IV. 3-years outcome of follow-up of patients with chronic obstructive pulmonary disease successfully treated by percutaneous coronary intervention due to acute coronary syndrome. Kardiologiia. (2020) 60(9):84–91. 10.18087/cardio.2020.9.n1263 33131479

[15] Gök G Kilic S Sinan ÜY Kılıç S Turkoglu E Kemal H. Epidemiology and clinical characteristics of hospitalized elderly patients for heart failure with reduced, mid-range and preserved ejection fraction. Heart Lung. (2020) 49(5):495–500. 10.1016/j.hrtlng.2020.03.023 32434698

[16] Plakht Y Gilutz H Shiyovich A. Decreased admission serum albumin level is an independent predictor of long-term mortality in hospital survivors of acute myocardial infarction. Soroka acute myocardial infarction II (SAMI-II) project. Int J Cardiol. (2016) 219:20–4. 10.1016/j.ijcard.2016.05.067 27257851

[17] Marco V Héctor B Byrne RA Valgimigli M Bueno H Collet J-P. 2017 ESC focused update on dual antiplatelet therapy in coronary artery disease developed in collaboration with EACTS. Eur J Cardiothorac Surg. (2018) 53(1):34–78. 10.1093/ejcts/ezx334 29045581

[18] Costa F van Klaveren D James S Heg D Räber L Feres F. Derivation and validation of the predicting bleeding complications in patients undergoing stent implantation and subsequent dual antiplatelet therapy (PRECISE-DAPT) score: a pooled analysis of individual-patient datasets from clinical trials. Lancet. (2017) 389(10073):1025–34. 10.1016/S0140-6736(17)30397-5 28290994

[19] Jonik S Skrobucha A Huczek Z Kochman J Opolski G Grabowski M. Impact of anemia on clinical outcomes in patients with multivessel coronary artery disease treated with percutaneous coronary intervention. Adv Interv Cardiol. (2024) 20(4):393–400. 10.5114/aic.2024.144778 PMC11783259 39897000

[20] Li J Liu F-H Guo J Yu Y-F Li C-Q. Retrospective analysis of renal prognosis in elderly coronary artery disease patients complicated with renal insufficiency. Aging (Albany NY). (2021) 13(19):22856–66. 10.18632/aging.203579 34606471 PMC8544318

